# Oxidized phospholipids facilitate calcific aortic valve disease by elevating ATF4 through the PERK/eIF2α axis

**DOI:** 10.18632/aging.204875

**Published:** 2023-07-17

**Authors:** Xiaohua Zhu, Linjie Yang, Xu Han, Chen Huang, Gongcheng Huang, Tingju Wei, Liliang Shu, Jing Xu

**Affiliations:** 1 Department of Cardiovascular Surgery, The First Affiliated Hospital of Zhengzhou University, Zhengzhou 450000, P.R. China

**Keywords:** calcific aortic valve disease, oxidized phospholipids, ATF4, valvular interstitial cells, aortic valve calcification, osteogenic differentiation, macrophages, PERK/eIF2α pathway

## Abstract

In this study we sought to analyze the critical role of oxidized phospholipid (OxPL) in the progression of calcific aortic valve disease (CAVD) with the involvement of activating transcription factor 4 (ATF4). Differentially expressed genes related to CAVD were identified using bioinformatics analysis. Expression of ATF4 was examined in mouse models of aortic valve calcification (AVC) induced by the high cholesterol (HC) diet. Valvular interstitial cells (VICs) were then isolated from mouse non-calcified valve tissues, induced by osteogenic induction medium (OIM) and co-cultured with OxPAPC-stimulated macrophages. The effect of OxPLs regulating ATF4 on the macrophage polarization and osteogenic differentiation of VICs was examined with gain- and loss-of-function experiments in VICs and *in vivo*. In aortic valve tissues and OIM-induced VICs, ATF4 was highly expressed. ATF4 knockdown alleviated the osteogenic differentiation of VICs, as evidenced by reduced expression of bone morphogenetic protein-2 (BMP2), osteopontin (OPN), and osteocalcin. In addition, knockdown of ATF4 arrested the AVC *in vivo*. Meanwhile, OxPL promoted M1 polarization of macrophages and mediated osteogenic differentiation of VICs. Furthermore, OxPL up-regulated ATF4 expression through protein kinase R-like endoplasmic reticulum kinase (PERK)/eukaryotic translation initiation factor 2 subunit alpha (eIF2α) pathway. In conclusion, OxPL can potentially up-regulate the expression of ATF4, inducing macrophages polarized to M1 phenotype, osteogenic differentiation of VICs and AVC, thus accelerating the progression of CAVD.

## INTRODUCTION

Calcific aortic valve disease (CAVD) is a common cardiovascular disorder, predominantly affecting the elderly population and therefore posing a large burden worldwide [1, 2]. The pathological process of CAVD involves endothelial dysfunction and injury, extracellular matrix degradation, aberrant matrix deposition, fibrosis, mineralization, inflammation, lipid accumulation and neo-angiogenesis [3]. The mechanisms underlying the pathogenesis of CAVD remain elusive, and this disease lacks effective drugs [4]. There is an unmet need to investigate the molecular mechanisms of CAVD in face of high morbidity and mortality [5]. In this context, identification of possible future molecular targets may open up new opportunities for the prevention and management of this debilitating disease.

Oxidized phospholipid (OxPL) exerts proinflammatory features and can induce calcification of vascular cells, showing a key role in the development of CAVD [6, 7]. Strategies aimed at suppressing the proinflammatory effect of OxPL may prevent the progression of this disease [8]. OxPL is the product of lipid oxidation, which is present on the oxidized low-density lipoproteins and apoptotic cell membranes [9]. Interaction between oxidized lipids with pattern recognition receptors and activated inflammasome has been reported to polarize macrophages toward an inflammatory M1 phenotype [10]. In the calcific aortic valves, M1 phenotype is the major macrophage subsets and polarization of macrophages to M1 phenotype drives aortic valve calcification (AVC) [11]. Additionally, OxPL can induce elevation in the expression of activating transcription factor 4 (ATF4) in several types of endothelial cells [12]. ATF4 is a stress-induced transcription factor that aids in mediating stress responses in mammalian cells [13]. A recent study has confirmed the abundant expression of ATF4 in CAVD samples while its down-regulation can inhibit the osteogenic differentiation of valvular interstitial cells (VICs) and the subsequent AVC [14]. Indeed, previous investigation on CAVD has highlighted that valvular calcification process is consistent with the osteogenic process, as a subpopulation of VICs can spontaneously differentiate into osteoblast-like cells in calcified valves, and create nodules containing mineralized hydroxyapatite matrix *in vitro* [15]. Of note, although the critical roles of OxPL and ATF4 in CAVD have been previously unfolded, the interaction between the two in CAVD has been rarely reported. To address this issue, the present study aimed to investigate the regulatory relationship between OxPL and ATF4 in the development of CAVD, in hope of furthering the understanding of the regulatory role of OxPL and macrophages in the pathogenesis of CAVD, providing a new theoretical insight on the pathogenesis of CAVD and aiding in the development of more effective therapeutic targets against CAVD.

## RESULTS

### OxPL-induced macrophage polarization promotes osteogenic differentiation of VICs

Recent studies have shown that apolipoprotein carries proinflammatory and procalcifying OxPL, which promotes the occurrence of CAVD. OxPL can up-regulate the expression of ATF4 [12, 16]. OxPL can promote the formation of valve calcification in aortic stenosis [17]. It can also induce macrophage polarization, and stimulate macrophage infiltration and inflammation [18, 19]. Therefore, we speculate that OxPL can promote AVC by inducing macrophage polarization.

**Figure 1:**
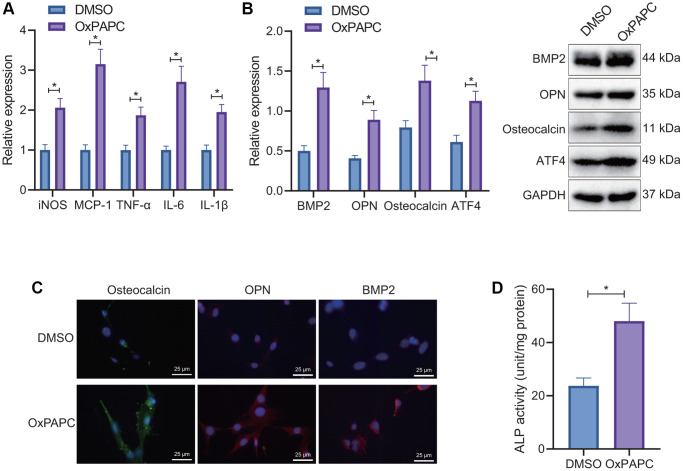
**Regulation of OxPL-induced macrophages on osteogenic differentiation in VICs.** (**A**) OxPL was used to stimulate mouse macrophages RAW264.7, and RT-qPCR to detect the expression of M1 macrophage related factors. (**B**) Mouse macrophages RAW264.7 stimulated by OxPL were co-cultured with VICs for 7 days, and Western blot was used to detect the expression of ATF4 and osteogenic factors BMP2, OPN, and osteocalcin in VICs. (**C**) IF detection of osteogenic factors BMP2, OPN, and osteocalcin expression in VICs. (**D**) Mouse macrophages RAW264.7 stimulated by OxPL were co-cultured with VICs for 7 days to detect the activity of ALP in VICs. ^*^p < 0.05. Cell experiments were repeated three times independently. Measurement data were expressed as mean ± standard deviation. Data obeying normal distribution and homogeneity of variance between two groups were analyzed using unpaired *t*-test.

First, we used oxidized 1-palmitoyl-2-arachidonoyl- sn-glycero-3-phosphorylcholine (OxPAPC) (one of OxPLs) to treat macrophages for 6 hours. Reverse transcription-quantitative polymerase chain reaction (RT-qPCR) detection found that OxPAPC treatment contributed to significant increases in the expression of inducible nitric oxide synthase (iNOS), monocyte chemoattractant protein-1 (MCP-1), tumor necrosis factor-α (TNF-α), interleukin-6 (IL-6), and interleukin-1β (IL-1β), indicating that OxPL promoted macrophage M1 polarization and the release of pro-inflammatory factors (Figure 1A). After co-culture of OxPL-induced macrophages with VICs for 7 days, Western blot and immunofluorescence (IF) results showed that the expression of osteogenic factors ATF4, bone morphogenetic protein-2 (BMP2), osteopontin (OPN), and osteocalcin was significantly increased by OxPAPC treatment (Figure 1B, 1C). Meanwhile, a marked increase in the activity of alkaline phosphatase (ALP) was noted after OxPAPC treatment (Figure 1D).

Thus, OxPL can promote osteogenic differentiation of VICs by inducing M1 polarization of macrophages. However, the mechanism by which OxPL-polarized macrophages promote the osteogenic differentiation of VIC is unclear. Therefore, we conducted a genomic analysis.

### ATF4 is highly expressed in CAVD samples

**Figure 2:**
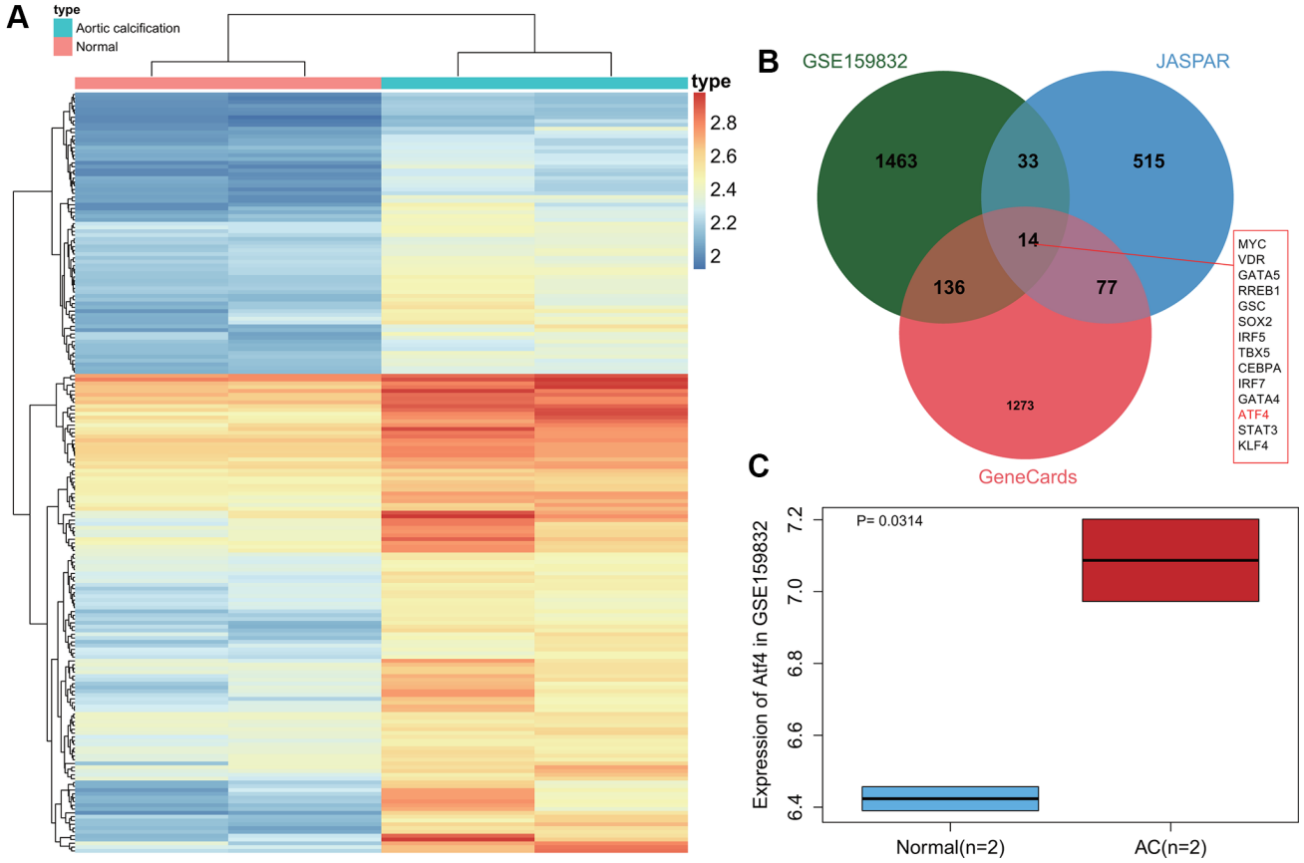
**Abundant expression of ATF4 in CAVD samples.** (**A**) A heat map of differentially expressed gene expression in 2 normal samples and 2 CAVD samples in the GSE159832 dataset. (**B**) Venn diagram of the differentially expressed genes in the GSE159832 dataset, CAVD-related transcription factors from the JASPAR database, and the top 1500 CAVD-related genes from the GeneCards database. (**C**) A box plot of ATF4 expression in 2 normal samples (blue box) and 2 CAVD samples (red box) in the GSE159832 dataset. *p* = 0.0314. ^*^p < 0.05.

First, differential analysis on the CAVD-related dataset GSE159832 revealed 1646 genes that were significantly up-regulated in calcified aortic valve tissues (Figure 2A). In addition, 639 transcription factors related to CAVD were obtained from the JASPAR database, and the top 1500 genes related to CAVD were obtained from the GeneCards database. Following intersection analysis, 14 transcription factors were found to be more important in the occurrence of CAVD (Figure 2B). Published literature has indicated that ATF4 is significantly highly expressed in CAVD samples, while its poor expression can inhibit the osteogenic differentiation of VICs [14, 20]. Here, a box plot based on the GSE159832 dataset further confirmed the elevated ATF4 expression in CAVD (Figure 2C). The above results indicate that ATF4 is highly expressed in CAVD samples, and that it may be involved in the occurrence of CAVD.

### Osteogenic differentiation of VICs is associated with high expression of ATF4

**Figure 3:**
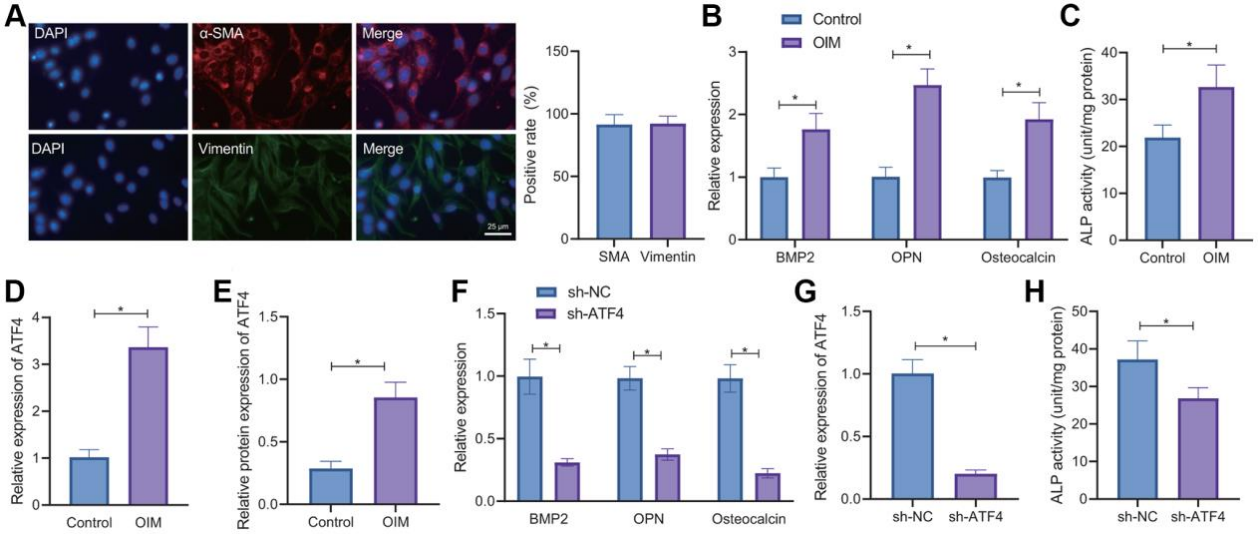
**ATF4 knockdown delays the osteogenic differentiation of VICs.** (**A**) IF staining of α-SMA and vimentin proteins in the VICs isolated from non-calcified valve tissues. (**B**) RT-qPCR detection of BMP2, OPN, and osteocalcin expression in the VICs induced by OIM and normal culture medium. (**C**) ALP activity in the OIM-induced VICs. (**D**) RT-qPCR detection of ATF4 expression in the VICs induced by OIM. (**E**) Western blot analysis of ATF4 protein in the OIM-induced VICs. (**F**) RT-qPCR detection of ATF4 expression in the OIM-induced VICs treated with sh-ATF4. (**G**) RT-qPCR detection of BMP2, OPN, and osteocalcin expression in the OIM-induced VICs treated with sh-ATF4. (**H**) ALP activity in the OIM-induced VICs treated with sh-ATF4. ^*^p < 0.05. Cell experiments were repeated three times independently. Measurement data were expressed as mean ± standard deviation. Data obeying normal distribution and homogeneity of variance between two groups were analyzed using unpaired *t*-test.

Occurrence of CAVD is closely related to the osteogenic differentiation of VICs [21]. Therefore, we isolated VICs from non-calcified valve tissues. IF results showed the presence of a large amount of α-smooth muscle actin (α-SMA) and vimentin proteins in VICs (Figure 3A), demonstrating the successful isolation of VICs. In addition, RT-qPCR results exhibited higher expression of BMP2, OPN and osteocalcin in the VICs induced by osteogenic induction medium (OIM) compared with the normal culture medium (Figure 3B). Moreover, ALP activity in the OIM-induced VICs was also increased (Figure 3C). These results explain that OIM can promote osteogenic differentiation of VICs.

As shown in Figure 3D, 3E, ATF4 mRNA and protein expression was elevated in the OIM-induced VICs. Silencing of ATF4 in the OIM-induced VICs led to a decline in the ATF4 mRNA expression (Figure 3F). Further, silencing of ATF4 could decrease the expression of BMP2, OPN, and osteocalcin (Figure 3G) as well as the ALP activity in OIM-induced VICs (Figure 3H). Taken together, overexpression of ATF4 may be accountable for the osteogenic differentiation of VICs.

### Knockdown of ATF4 arrests AVC in mice

**Figure 4:**
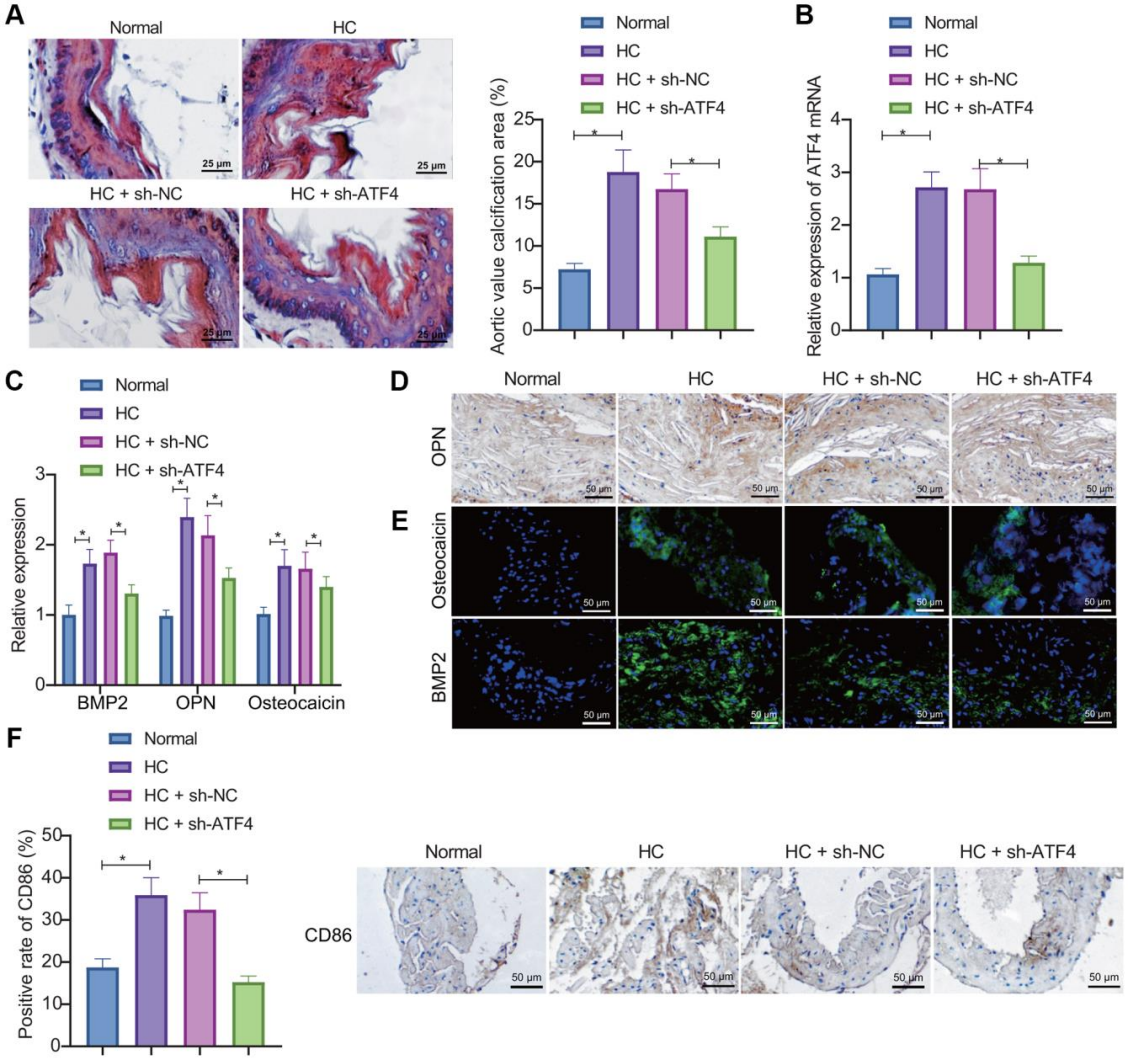
**ATF4 silencing represses AVC in mice.** (**A**) ARS staining of aortic valve leaflet calcification in HC-induced mice treated with sh-ATF4. (**B**) Expression of ATF4 in the aortic valve tissue of HC-induced mice treated with sh-ATF4 detected by RT-qPCR. (**C**–**E**) Expression of BMP2, OPN, and osteocalcin in the aortic valve tissue of HC-induced mice treated with sh-ATF4 detected by RT-qPCR, IHC, and IF. (**F**) IHC of CD86 protein in the aortic valve tissue of HC-induced mice treated with sh-ATF4. ^*^p < 0.05. *n* = 8. Measurement data were expressed as mean ± standard deviation. Data obeying normal distribution and homogeneity of variance among multiple groups were analyzed by one-way ANOVA with Tukey's post hoc tests.

We then focused on probing the effect of ATF4 on the AVC *in vivo*. First, RT-qPCR, immunohistochemistry (IHC), and IF were used to detect the expression of bone related factors in the aortic valve tissue. The results showed that the expression of BMP2, OPN, and osteocalcin increased in the aortic valve tissue of high cholesterol (HC)-induced mice, indicating the successful construction of the mouse model of AVC. Determination of ATF4 expression by RT-qPCR depicted that ATF4 expression was increased in the aortic valve tissue of HC-induced mice while treatment with sh-ATF4 led to opposite results (Figure 4B), demonstrating that the lentivirus indeed knocked down the expression of ATF4 in mice. Meanwhile, the expression of ATF4 was elevated during the occurrence of AVC. Alizarin red S (ARS) staining showed obvious calcification in the aortic valve leaflets in HC group, while ATF4 knockdown could markedly reduce the calcification area (Figure 4A). Besides, the up-regulated expression of BMP2, OPN, and osteocalcin in the aortic valve tissue of HC-induced mice could be reversed by sh-ATF4 (Figure 4C–4E). Furthermore, IHC results demonstrated increased expression of CD86 in the aortic valve tissue of HC-induced mice but further ATF4 silencing diminished the expression of CD86 (Figure 4F). Thereby, it can be concluded that AVC in HC-induced mice is associated with up-regulation of ATF4 expression and that the number of macrophages M1 increases during the concurrent onset.

### OxPL-polarized macrophages promote osteogenic differentiation of VICs by up-regulating ATF4

**Figure 5:**
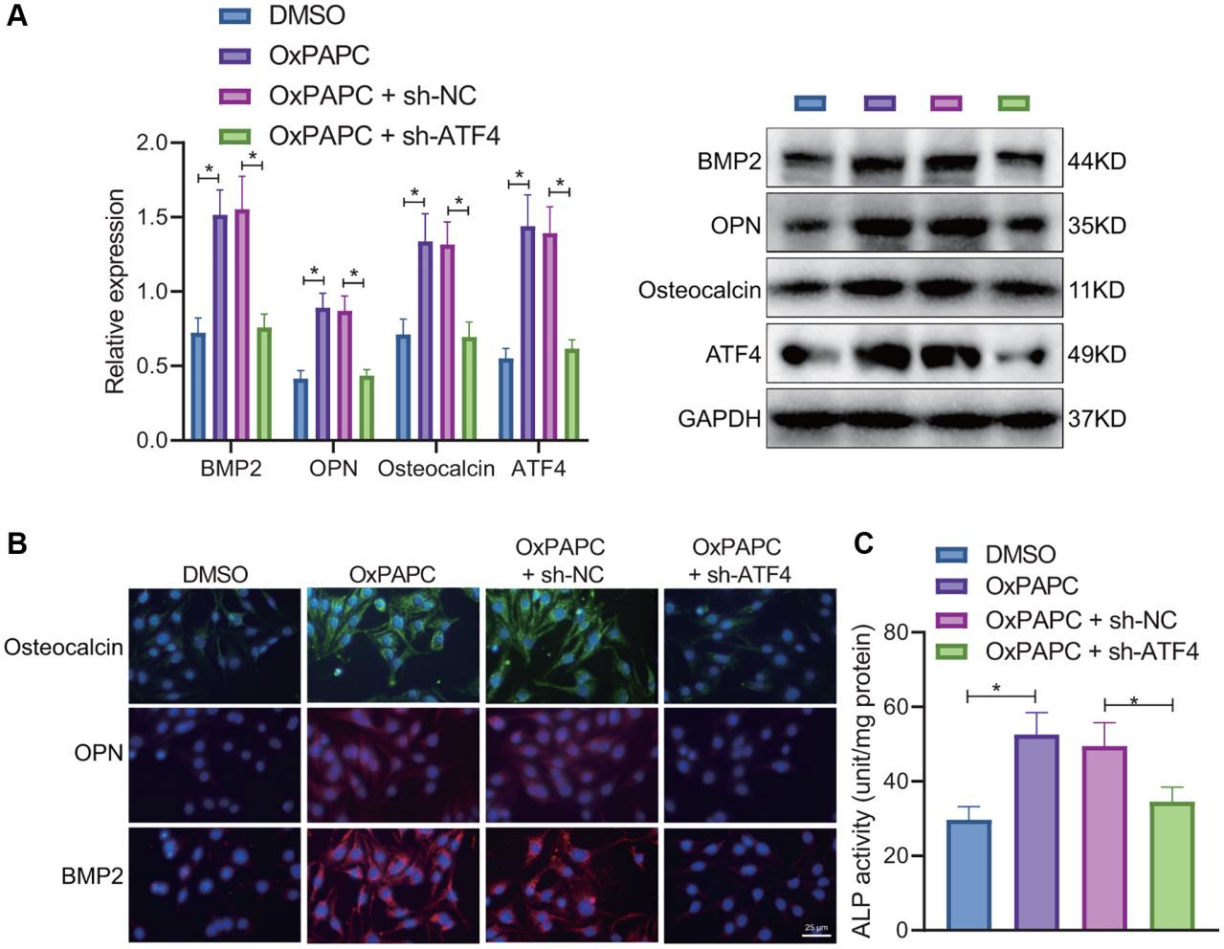
**OxPAPC-polarized macrophages promote osteogenic differentiation of VICs through ATF4.** OxPAPC- or DMSO- polarized macrophages were co-cultured with VICs transduced with sh-ATF4. (**A**) The protein expression of BMP2, OPN and osteocalcin detected by Western blot. (**B**) The protein expression of BMP2, OPN and osteocalcin detected by IF. (**C**) ALP activity determination. ^*^p < 0.05. *n* = 8. Cell experiments were repeated three times independently. Measurement data were expressed as mean ± standard deviation. Data obeying normal distribution and homogeneity of variance between two groups were analyzed using unpaired *t*-test. Data obeying normal distribution and homogeneity of variance among multiple groups were analyzed by one-way ANOVA with Tukey’s post hoc tests.

Furthermore, we verified the relationship between OxPL-polarized macrophages, ATF4, and osteogenic differentiation of VICs. Macrophages were treated with OxPAPC or DMSO for 6 hours and then co-cultured with VICs transduced with short hairpin RNA (sh)-ATF4 or sh-negative control (NC) for 7 days, and VICs were collected for testing. Western blot and IF results revealed that knockdown of ATF4 decreased the protein expression of BMP2, OPN, and osteocalcin in response to OxPAPC (Figure 5A, 5B). Meanwhile, the ALP activity in the presence of OxPAPC or OxPAPC-sh-NC was significantly higher than that upon OxPAPC and sh-ATF4 in combination (Figure 5C). The results suggest that OxPAPC-polarized macrophages enhance the expression of ATF4 to promote the osteogenic differentiation of VICs.

### OxPL up-regulates ATF4 expression through protein kinase R-like endoplasmic reticulum kinase (*PERK*)/eukaryotic translation initiation factor 2 subunit alpha (eIF2α) pathway

**Figure 6:**
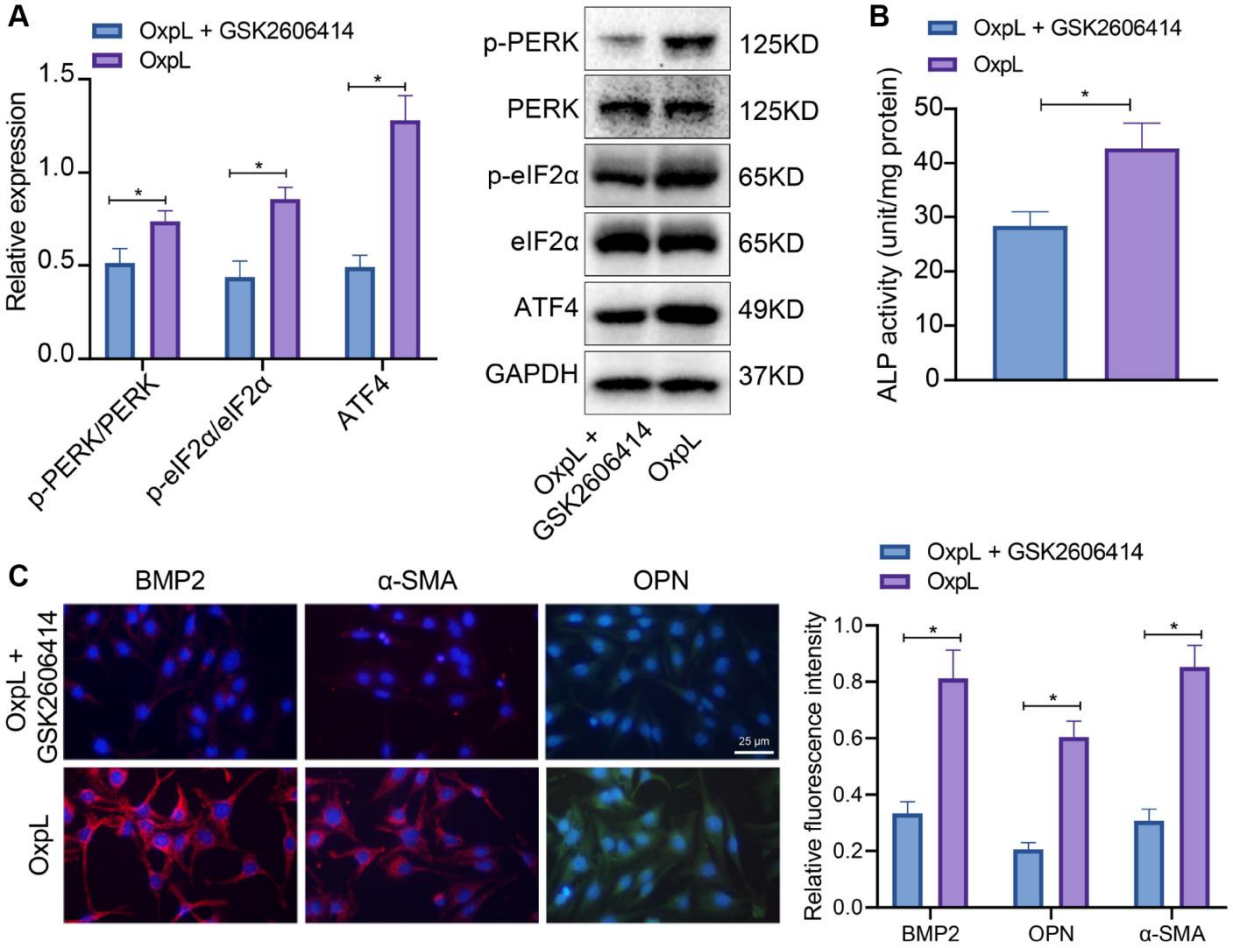
**OxPL up-regulates ATF4 expression through PERK/eIF2α pathway.** (**A**) OxPAPC polarized macrophages and VICs were co-cultured, followed by addition of PERK inhibitor, and the phosphorylation levels of PERK and eIF2α as well as ATF4 expression in VICs was detected by Western blot. (**B**) ALP activity determination. (**C**) BMP2, α-SMA and OPN expression in VICs was detected by IF. ^*^p < 0.05. Cell experiments were repeated three times independently. Measurement data were expressed as mean ± standard deviation. Data obeying normal distribution and homogeneity of variance between two groups were analyzed using unpaired *t*-test, while those among multiple groups were analyzed by one-way ANOVA with Tukey’s post hoc tests.

Combined with the literature and our above experiments, it is known that the occurrence of CAVD is closely related to the up-regulation of ATF4 expression in VICs, and the high expression of ATF4 is closely related to the M1 polarization of macrophage. However, the mechanism by which macrophage polarization promotes the high expression of ATF4 is still unclear. It has been reported that PERK/eIF2α/ATF4 is an important endoplasmic reticulum stress pathway [22] and closely related to inflammation [23]. Therefore, PERK inhibitor (GSK2606414) was added to the cell fluid of OxPL-polarized macrophages co-cultured with VICs to detect the expression of ATF4 and osteogenic differentiation-related proteins. It was found that the phosphorylation levels of PERK and eIF2α, ALP activity, as well as ATF4, BMP2, α-SMA and OPN expression were significantly increased in VICs co-cultured with OxPL-polarized macrophages. After GSK2606414 treatment, the phosphorylation levels of PERK and eIF2α, ALP activity, as well as ATF4, BMP2, α-SMA and OPN expression was significantly down-regulated (Figure 6A–6C). The aforesaid results suggest that OxPL induces the M1 polarization of macrophages, and polarized macrophages promote the expression of ATF4 through PERK/eIF2α pathway, which exacerbates AVC.

## DISCUSSION

Herein, we attempted to investigate the role of OxPL in CAVD by regulating ATF4, and the current findings unveil that OxPL can potentially up-regulate the expression of ATF4, thus promoting LPS-induced M1 polarization of macrophages, enhancing the osteogenic differentiation of VICs and formation of AVC, and eventually leading to the initiation of CAVD.

Our work demonstrated robustly induced ATF4 in CAVD samples and that knockdown of ATF4 could arrest the osteogenic differentiation of VICs, as evidenced by the decreased expression of BMP2, OPN, and osteocalcin as well as ALP activity. BMP2, OPN and ALP are osteogenic differentiation markers and their increased expression is associated with promotion of the osteogenic differentiation of VICs [11]. Importantly, the transform of VICs to an osteoblast-like phenotype is proved as the basic hallmark of triggered valvular calcification [24]. ATF4 has been a critical transcription factor for osteoblast function and ATF4 knockdown can lead to a drastic decrease in the osteoclast differentiation [25]. In addition, ATF4 is significantly induced in VICs from calcified aortic valve tissues, which can promote activation of osteoblastic differentiation of VICs and the resultant AVC [26]. Although previous studies have already unveiled the high expression of ATF4 and its role in CAVD, differently, in the present study we verified the up-regulated ATF4 in CAVD through independent bioinformatics methods, demonstrated that knockdown of ATF4 could delay AVC in HC-induced mice, and confirmed that OxPL-polarized macrophages promoted osteogenic differentiation in CAVD by up-regulating ATF4 through PERK/eIF2α axis. OxPL has been shown to drive AVC and accelerate the progression of aortic stenosis, which are associated with the pro-osteogenic effects of OxPL on VICs [17]. In addition, macrophages can alter their gene expression and function drastically upon exposure to OxPL [8]. For instance, OxPL stimulation is capable of augmenting the expression of genes involved in macrophage infiltration and inflammation [19]. Also, the phenotypic polarization of macrophages can be influenced by bioactive plaque lipids and OxPL induces the expression of inflammatory genes in M1 macrophages [10]. Previous literature has highlighted that OxPL can up-regulate the protein expression of ATF4 in human endothelial cells [16]. As previously reported, PERK/eIF2α/ATF4 is an important endoplasmic reticulum (ER) stress pathway [22] and closely correlated with inflammation [23]. ATF4 can modulate the ER stress response induced by loperamide and then mediate the corresponding autophagy process [27]. Similarly, ATF4 is able to mediate the invasion process of tumor cells during ER stress through PERK/eIF2α/ATF4/CHOP axis [28]. More importantly, a large number of studies have shown that ATF4 is very important for cells to cope with ER stress response induced by ischemia/reperfusion and tunicamycin [29, 30]. These lines of evidence suggest inactivating the OxPL/PERK/eIF2α/ATF4 axis may slow AVC and the CAVD progression.

**Figure 7:**
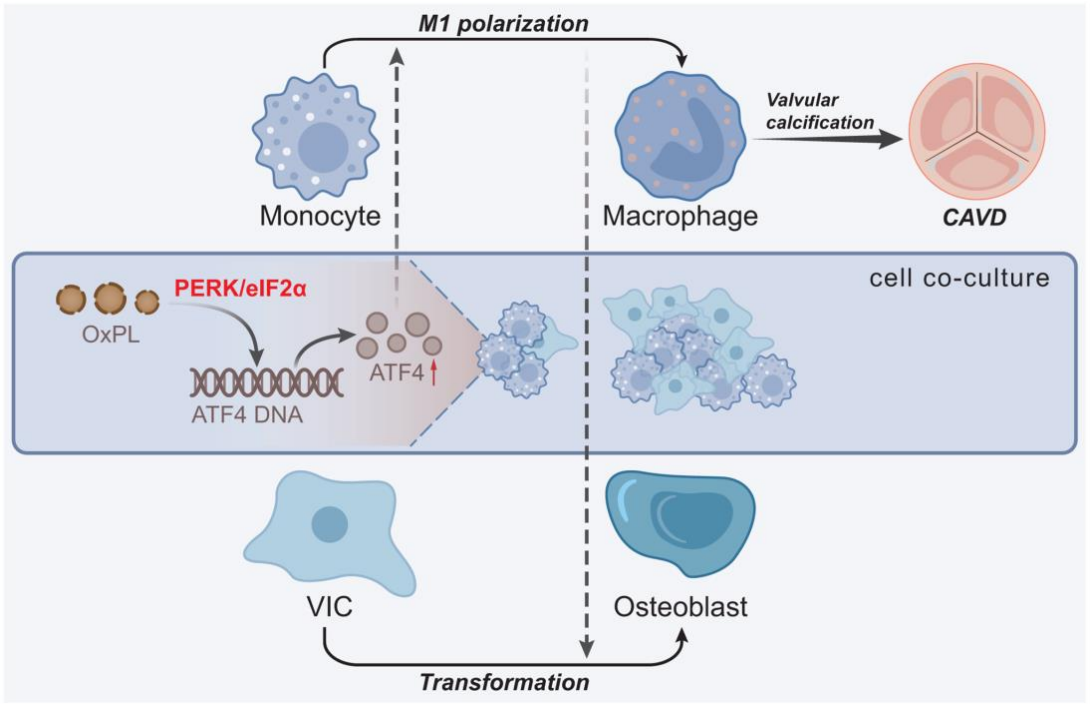
**Molecular mechanism underlying the role of OxPL in CAVD.** OxPL promotes the M1 polarization of macrophages by up-regulating the expression of ATF4, thereby enhancing the osteogenic differentiation of VICs and formation of AVC, ultimately promoting the occurrence of CAVD.

Taken together, OxPL contributed to AVC and the resultant CAVD. The underlying regulatory mechanism is that OxPL exerts its function through functioning as a promoting factor for ATF4 and PERK/eIF2α pathway, thus augmenting the M1 polarization of macrophages and the osteogenic differentiation of VICs (Figure 7). This study provides novel insights into the regulatory relationship between OxPL and ATF4 in the progression of CAVD. Therefore, this study contributes to a better understanding of the molecular mechanisms underlying the pathogenesis of CAVD and highlights the potential of targeting the OxPL-ATF4 axis as a therapeutic strategy for the treatment of this disease. However, there are still several limitations in the study. Firstly, the study was conducted in mouse VICs *in vitro* and HC-induced mice *in vivo*, which may not accurately reflect the complexity of human physiology and disease pathogenesis. Further exploration is warranted to confirm the results in human subjects. Secondly, further animal experiments with animal gross specimens are needed for validation. Thirdly, in spite of the discovery of ATF4 as a potential therapeutic target in this study, more research is needed to develop effective and safe treatments for CAVD patients.

## METHODS

### *In silico* analysis

CAVD-related mRNA expression dataset GSE159832 (2 normal samples, 2 ApoE samples, and 2 chronic kidney disease samples) was retrieved from the Gene Expression Omnibus database. In the current study, we only selected normal and ApoE samples for analysis. Differential analysis was completed with the help of R language “limma” package with |logFC| > 0.05 and *p* < 0.05 as the threshold to screen significantly up-regulated genes. Human transcription factors were retrieved from the JASPAR database, and the top 1500 genes related to CAVD were obtained from the GeneCards database, followed by intersection analysis to determine the key transcription factors.

### Establishment of high cholesterol (HC)-induced mouse AVC models

Thirty-two males ApoE−/− mice (aged 6–8 weeks old, Beijing Biocytogen Co., Ltd., Beijing, China) were enrolled in this study. Eight randomly selected mice were fed a normal diet and the remaining mice were fed a HC diet (0.2% cholesterol and 20% fat). The HC-fed mice were further injected with 100 μL lentivirus (Shanghai Genechem Co., Ltd., Shanghai, China) carrying sh-NC and sh-ATF4 at a titer of 7.6 × 10^7^ infectious units via caudal vein, with 8 mice for each treatment. After twenty weeks of HC feeding, the mice were euthanized, and the aortic valve tissue was removed for subsequent analysis. ARS staining was used to determine the calcification area of the aortic valve leaflets.

### Isolation and culture of mouse VICs

VICs were isolated from non-calcified valve tissues of normal mice. The valve leaflets were detached in type II collagenase solution (2.0 mg/mL) at 37°C for 30 minutes. The endothelial cells were removed by vortexing, whereupon the leaflets were detached in fresh type II collagenase solution (2.0 mg/mL) at 37°C for 2 hours. Thereafter, the test tube was vigorously rotated to discard the undetached leaflets. The suspension was centrifuged at 500 g for 5 minutes to collect the separated cells. The cells were then resuspended in DMEM (penicillin G, streptomycin and 10% FBS), plated in a six-well plate, and cultured in a 5% CO_2_ incubator at 37°C. IF was used to detect mesenchymal cells marker proteins vimentin and α-SMA for VIC identification. When the cells reached 70–90% confluence, they were sub-cultured. Cells at passage 3–7 were used for the experiment. VICs were co-cultured with OxPAPC-stimulated macrophages for 7 days, and the expression of related factors in VICs was detected by RT-qPCR.

VICs were then transduced with lentivirus carrying sh-NC and sh-ATF4. All lentivirus contained GFP fluorescence and was purchased from Genechem. Before experiments, lentivirus was diluted with phosphate buffered saline (PBS) into different gradients, and then used to transduce the cells in the 96-well plate. After 24 hours, the GFP fluorescence intensity of each virus gradient was observed under a fluorescence microscope. The most appropriate titer was selected for formal transduction. The cells were seeded in a 24-well plate at a density of 5 × 10^4^ cells/well. Upon logarithmic growth phase, the cells were added with virus solution and 10 μg/mL Polybrene (H8761, Beijing Solarbio Science and Technology Co., Ltd., Beijing, China) to promote transduction efficiency. After 16–24 hours, the solution was changed. After 72 hours, 1 μg/mL puromycin (A1113803, Invitrogen Inc., Carlsbad, CA, USA) was added for cell selection. When the cells grew stably, the interference efficiency was tested by RT-qPCR.

### Culture and polarization of mouse macrophages

Mouse macrophages RAW 264.7 purchased from ATCC (Manassas, VA, USA) were cultured in DMEM containing 10% FBS (Hyclone, Logan, UT, USA) in a 5% CO_2_ incubator at 37°C. The medium was renewed every 48 hours. In order to activate the macrophages with M1 phenotype, RAW 264.7 cells were stimulated with 130 μM OxPAPC. After 24 hours, the cells were collected to detect M1 macrophage markers.

### Osteogenic differentiation induction of VICs

When reaching 70–80% confluence, VICs were treated with osteogenic induction medium (OIM) for 4 days to induce osteoblast differentiation. OIM consisted of a complete growth medium supplemented with 50 ng/mL bone morphogenetic protein 2, 50 mg/mL ascorbic acid-2-phosphate, 10 nmol/L dexamethasone and 10 mmol/L b-Glycerophosphate.

### ALP activity detection

ALP activity was measured to quantify the degree of osteogenic differentiation of VICs. The ALP activity of cells was evaluated at 405 nm using the p-nitrophenol phosphate substrate kit (Sigma-Aldrich, St. Louis, MO, USA). The ALP value (units/ng protein) was normalized to the protein value using the Bio-Rad DC protein assay (Bio-Rad, Hercules, CA, USA).

### ARS staining

After 14 days of osteogenic differentiation, ARS staining was performed to test matrix mineralization deposits, which appeared in the later stages of bone formation. The matrix calcification stained with ARS appeared as red deposits.

### RNA isolation and quantitation

Total RNA was extracted from cells using the TRIzol reagent (15596026, Invitrogen, USA), 1 μg of which was then reversely transcribed into complementary DNA (cDNA) using a First Strand cDNA Synthesis kit (K1622, Fermentas, USA). RT-qPCR was conducted using the Fast SYBR Green PCR kit (Applied Biosystems Inc., Carlsbad, CA, USA) on the ABI PRISM 7300 RT-PCR system (Applied Biosystems). Three duplicated wells were set for each sample. With glyceraldehyde-3-phosphate dehydrogenase serving as internal control, the fold changes were calculated by means of relative quantification (2^−ΔΔCt^ method). All primers were procured from Sangon Biotech Co., Ltd. (Shanghai, China) and the sequences are shown in Supplementary Table 1.

### Western blot analysis

Total protein extracts were separated by 10% SDS-PAGE and transferred onto a polyvinylidene fluoride membrane. Next, the membrane was blocked with 5% bovine serum albumin for 2 hours and underwent overnight incubation at 4°C with primary antibodies (Supplementary Table 2). The following day, the membrane was incubated with horseradish peroxidase (HRP)-labeled secondary antibody at room temperature for 1 hour. Afterwards, the immunocomplexes on the membrane were visualized using enhanced chemiluminescence reagent (BM101, Biomiga, San Diego, CA, USA). Image exposure was performed on BioSpectrum 600 imaging system (Ultra-Violet Products, Cambridge, UK). Image J software was used for quantitative analysis.

### IHC

The collected paraffin sections of mouse aortic valve tissues were placed in an incubator, stored at 60°C for 2 hours, and then immersed in water. The sections were rehydrated with different concentrations of ethanol (100, 95, 85, 70%) and deionized water, then soaked in citric acid buffer (0.01 mol/L, pH 6.0), and heated at 95–100°C for 30 minutes. Following PBS washing, the sections were incubated with 0.5% Triton X-100 for 30 minutes and stained with biotin-streptavidin HRP detection system (ZSGB, Beijing, China). Thereafter, the sections were probed overnight at 4°C with primary antibodies against CD86 (1:200, ab220188, Abcam, Cambridge, UK) and OPN (1:500, ab218237, Abcam). The next day, the sections were re-probed with secondary antibodies (goat anti-rabbit, #3900, Cell Signaling Technology (CST); goat anti-mouse, ab150115, Abcam) for 1 hour, and observed under a microscope. The brown chromogen on the membrane indicated a positive immunoreaction. The image was visualized using Nikon Eclipse Ti microscope (Fukasawa, Japan) and processed with Nikon software.

### IF

The autoclaved cell slide was put into a 24-well plate where cells were seeded at a density of 5 × 10^4^ cells/well. After 16 hours, the cells adhering to the wall were then washed twice with PBS, fixed in 500 μL of 4% paraformaldehyde for 20 minutes, and permeabilized with 300 μL of 0.5% Triton X for 10 minutes. Thereafter, the cells were blocked with goat serum for 1 hour and probed overnight with primary antibodies prepared with goat serum against vimentin (1:100, #5741, CST), α-SMA (1:500, #19245, CST), osteocalcin (1:100, ab198228, Abcam), BMP2 (1:100, ab284387, Abcam), and OPN (1:1000, ab218237, Abcam). The next day, the cells were re-probed with secondary antibodies prepared with PBS against goat anti-rabbit immunoglobulin G (IgG) (#2985, CST) and goat anti-mouse IgG (#4417, CST) in the dark for 1 hour, followed by 4′6-diamidino-2-phenylindole (DAPI) staining for 15 minutes. Finally, the cells were sealed with fluorescence quenching agent and photographed with a Nikon confocal microscope.

Mouse aortic valve tissue sections were dewaxed, hydrated, and subjected to antigen retrieval in sodium citrate buffer for 5 minutes, followed by incubation for 1 hour in a solution containing 5% goat serum to prevent nonspecific binding. The sections were incubated with osteocalcin (1:100, ab198228, Abcam) and BMP2 (1:100, ab284387, Abcam) at 4°C overnight. Then, the sections were incubated with goat anti-rabbit IgG (Alexa Flug 488, 1:1000, ab150077, Abcam) secondary antibody, followed by DAPI treatment. Fluorescence images were taken with a Nikon confocal microscope.

### Statistical analysis

All data were processed using Statistical Product and Service Solutions 21.0 statistical software (IBM Corp. Armonk, NY, USA). Measurement data were expressed as mean ± standard deviation. Tests for normality and homogeneity of variance were conducted. Unpaired *t*-test was applied for data comparison while data among multiple groups were analyzed by one-way analysis of variance (ANOVA) with Tukey’s post hoc tests. Rank-sum tests were performed when the data did not show normal distribution or equal variance. *p* < 0.05 was considered statistically significant.

### Data availability

The datasets used and/or analysed during the current study are available from the corresponding author on reasonable request.

## Supplementary Materials

Supplementary Tables
