# Supplementary material for: Oxidized phospholipids facilitate calcific aortic valve disease by elevating ATF4 through the PERK/eIF2α axis
Source: Aging (Albany NY). 2023 Jul 17;15(14):6834–47. doi: 10.18632/aging.204875 (PMC10415544; doi:10.18632/aging.204875)
Supplement: Supplementary Tables [file aging-15-204875-s001.pdf]

SUPPLEMENTARY TABLES

Supplementary Table 1. Primer sequences for RT-qPCR.

Target	Sequences
Mmu-ATF4	Forward: 5′-CCTGAACAGCGAAGTGTTGG-3′<br>Reverse: 5′-TGGAGAACCCATGAGGTTTCAA-3′
Mmu-BMP2	Forward: 5′-GGGACCCGCTGTCTTCTAGT-3′<br>Reverse: 5′-TCAACTCAAATTCGCTGAGGAC-3′
Mmu-OPN	Forward: 5′-ATCTCACCATTCGGATGAGTCT-3′<br>Reverse: 5′-TGTAGGGACGATTGGAGTGAAA-3′
Mmu-Osteocalcin	Forward: 5′-CTGACCTCACAGATGCCAAGC-3′<br>Reverse: 5′-AGATGCGTTTGTAGGCGGTC-3′
Mmu-TNF-α	Forward: 5′-ACGGCATGGATCTCAAAGAC-3′<br>Reverse: 5′-AGATAGCAAATCGGCTGACG-3′
Mmu-IL-6	Forward: 5′-GTCCTTCCTACCCCAATTTCCA-3′<br>Reverse: 5′-TAACGCACTAGGTTTGCCGA-3′
Mmu-IL-1β	Forward: 5′-TGTGAAATGCCACCTTTTGA-3′<br>Reverse: 5′-GGTCAAAGGTTTGGAAGCAG-3′
Mmu-iNOS	Forward: 5′-CCAAGCCCTCACCTACTTCC-3′<br>Reverse: 5′-CTCTGAGGGCTGACACAAGG-3′
Mmu-MCP1	Forward: 5′-CCACTCACCTGCTGCTACTCA-3′<br>Reverse: 5′-TGGTGATCCTCTTGTAGCTCTCC-3′
Mmu-GAPDH	Forward: 5′-AGGTCGGTGTGAACGGATTTG-3′<br>Reverse: 5′-GGGGTCGTTGATGGCAACA-3′

Supplementary Table 2. Antibodies used for Western blot analysis.

Antibody	Molecular weight	Dilution ratio	Source and product number
ATF4	49 kDa	1:1000	CST, #11815
GAPDH	37 kDa	1:1000	CST, #2118
p-PERK/PERK	125 kDa	1:1000	Abcam, ab229912
P-eIF-2a/eIF-2a	65 kDa	1:2000	Abcam, ab169528
BMP2	44 kDa	1:1000	Abcam, ab284387
OPN	35 kDa	1:1000	Abcam, ab214050
Osteocalcin	11 kDa	1:10000	Abcam, ab133612
Anti-rabbit IgG (secondary antibody)		1:1000	CST, #7074
Anti-mouse IgG (secondary antibody)		1:1000	CST, #7076
